# A Molecular Perspective on the Potential Benefits of Metformin for the Treatment of Inflammatory Skin Disorders

**DOI:** 10.3390/ijms21238960

**Published:** 2020-11-25

**Authors:** Ji-Eun Chang, Min Sik Choi

**Affiliations:** 1 Lab of Pharmaceutics, College of Pharmacy, Dongduk Women’s University, Seoul 02748, Korea; jieun0515@dongduk.ac.kr; 2 Lab of Pharmacology, College of Pharmacy, Dongduk Women’s University, Seoul 02748, Korea

**Keywords:** metformin, inflammatory skin disorders, psoriasis, acanthosis nigricans, acne, hidradenitis suppurativa, allergic contact dermatitis

## Abstract

Due to its anti-hyperglycemic effect, metformin is the first-line medication for the treatment of type 2 diabetes, particularly in people who are obese. However, metformin is a drug with a very wide range of pharmacological properties and reports of its therapeutic effect on diseases including inflammation and cancer are increasing. Numerous research groups have reported that metformin has beneficial effects on a variety of inflammatory skin disorders including psoriasis, acanthosis nigricans, acne, hidradenitis suppurativa, and allergic contact dermatitis. According to these reports, in addition to the well-known action of metformin, that is, its anti-hyperglycemic effect, NF-kB inhibition and the resulting alteration to the cytokine network may be the potential targets of metformin. Its anti-hyperandrogenism effect has also been confirmed as the major action of metformin in some inflammatory skin diseases. Moreover, novel regulatory mechanisms, including autophagy and antioxidant processes, have been suggested as promising mechanisms of action for metformin in inflammatory skin disorders.

## 1. Introduction

Metformin is an oral anti-hyperglycemic drug that acts as an insulin sensitizer by reducing liver glucose production and increasing glucose utilization by muscle and fat cells [1]. Insulin resistance and compensatory hyperinsulinemia have been shown to be associated with an increased risk of developing type 2 diabetes, and hyperglycemia of diabetes is caused by impaired insulin secretion and insulin resistance. Insulin resistance (or decreased insulin sensitivity) is defined as the abnormal response of tissues to normal insulin levels. Therefore, improvements in blood sugar levels result in a slight decrease in serum insulin levels, which improves hyperinsulinemia. Metformin is the first line of treatment for patients with type 2 diabetes, especially those who are obese, because it has been shown to enhance clinical outcomes and quality of life for these people and to decrease their micro and macro-vascular problems [2,3].

Metformin has also showed lipid-lowering actions that are associated with reducing fatty liver, such as reducing serum triglyceride and free fatty acid concentrations, slightly reducing serum low-density lipoprotein (LDL) cholesterol levels, and increasing serum high-density lipoprotein (HDL) cholesterol levels [4]. In addition, recent studies have shown that metformin has an anti-platelet aggregation effect, which reduces the rate of production of the final glycated end products and reduces cellular oxidation reactions, and demonstrates its antioxidant effects [1]. These findings regarding the beneficial effects of metformin on cholesterol level and platelet aggregation, along with the previously mentioned blood sugar control effects, clearly demonstrate the vaso-protective effects of metformin. The most common side effects of metformin are gastrointestinal (i.e., anorexia, vomiting, nausea, abdominal discomfort, and diarrhea), which occur in up to 20% of patients.

At the molecular level, metformin regulates 5′-adenosine monophosphate-activated protein kinase (AMPK) through liver kinase B1 (LKB1). As LKB1 is a tumor suppressor protein, AMPK activation through LKB1 may play an important role in preventing cancer cell growth. In a case-controlled and cohort study of people with type 2 diabetes, metformin treatment was associated with reduced cancer risk and cancer mortality [5,6]. Although preclinical and in vitro study data have confirmed the anticancer activity of metformin against several types of cancer, the underlying mechanism of action for how metformin exerts anticancer activity is not yet fully understood.

Thus, metformin has a wide range of pharmacological properties. In fact, it is also applied to inflammatory skin diseases such as psoriasis, acne, and allergic contact dermatitis, i.e., non-diabetic situations. In fact, metformin has received a great deal of interest because it has been included in clinical trials over the last three years for inflammatory skin diseases such as hidradenitis suppurativa, acanthosis nigricans, and acne, and it has demonstrated positive effects [7,8,9]. This review aims to summarize the action of metformin in inflammatory skin diseases and to find its potential mechanism of action. The actions of metformin in the various inflammatory skin disorders that are covered in this review paper are summarized in Table 1.

## 2. Potential Molecular Mechanisms of Action for Metformin

The potential mechanisms of the anti-inflammatory effects of metformin on skin have not yet been fully identified; however, several studies have been conducted recently and the results suggest various new possibilities.

The anti-inflammatory actions of metformin have been reported in a variety of cell types. First, metformin has been reported to reduce the production of nitric oxide (NO), prostaglandin E2 (PGE2) and pro-inflammatory cytokines, such as IL-1β, IL-6, and TNF-α, through inhibition of the nuclear factor kappa-light-chain-enhancer of activated B cells (NF-κB) activation in macrophages [10,11]. The cytokines mentioned here do not only function as final products of inflammation, but crosstalk with each other’s signaling systems while mediating various skin inflammatory processes. In other words, it is necessary to go beyond the stage of verifying the effect of each cytokine, and to study the overall change in the cytokine network in inflammatory conditions. Meanwhile, the protein expression level of 11β-hydroxysteroid dehydrogenase type 1 (11β-HSD1), which regenerates active glucocorticoids, was elevated in adipose tissue of people with obesity and metabolic disorders, which is associated with inflammation [12]. Another study reported that metformin inhibited pro-inflammatory cytokine-induced 11β-HSD1 expression in human adipocytes through inhibition of the NF-κB pathway [13]. It has also been reported that 11β-HSD1 knockdown abrogates the response to pro-inflammatory cytokines in skin keratinocytes [14], and it is very likely that NF-κB is involved in this process as well. Together, the above studies suggest that NF-κB inhibition by the activation of AMPK plays an important role in metformin’s anti-inflammatory action and they provide an opportunity to discover new mechanisms of action.

In addition, poly [ADP-ribose] polymerase 1 (PARP-1) participates as a co-activator for NF-κB-mediated transcription, activating the pro-inflammatory pathway associated with p38 MAPK and c-Jun N-terminal kinase (JNK), and inhibiting anti-inflammatory function by Bcl-6 [15,16]. In a recent study, NAD-dependent deacetylase sirtuin-1 (SIRT1) was reported to regulate inflammation and apoptosis through LKB1/AMPK-dependent pathways in bovine retinal capillary endothelial cells (BREC) and in the retina of diabetic mice, and metformin activates the SIRT1/LKB1/AMPK pathway by inhibiting reactive oxygen species (ROS)/PARP signaling [17]. Meanwhile, metformin in rat lung tissue was reported to inhibit not only ROS production but also allergic eosinophilic inflammatory reactions through AMPK activation [18,19]. These reports suggest a potential mechanism of action for metformin in intracellular stress situations involving ROS production.

## 3. Inflammatory Skin Disorders

Diverse immune pathways in the skin have been determined over the years. These findings have changed our clinical practice by being interpreted into targeted therapies for inflammatory skin disorders. The skin is recognized as the largest organ of the human body that defends the body against the environment with an epithelial barrier consisting of numerous immune cells such as dendritic cells, macrophages, helper T cells (Th cells), cytotoxic T cells (Tc cells), B cells, lymphoid cells and granulocytes [20]. As these cells participate in various inflammatory processes when exposed to pathogens or allergens, it is generally considered that they are responsible for chronic inflammatory skin disorders. Here, the role of the key players in inflammatory skin disease will be reviewed.

First, the innate immune system is essential for preventing skin infections and eradicating pathogenic bacteria, and it plays a very important role in skin healing. Once it recognizes bacteria or viruses, the inflammatory cascade associated with the release of cytokines, the mobilization of immune cells, and the production of antibacterial peptides (AMPs) and interferon-stimulating genes (ISGs) are all initiated. The adaptive immune system induces the creation of antigen-specific immunity and immune memory through tightly regulated interactions between antigen-presenting cells and B/T lymphocytes [21]. The antigen specificity of B and T lymphocytes originates from antigen receptors encoded by genes, which are rearranged and reassembled to produce diversity. Research in recent years has found that T cells can be characterized by their unique Ag specificity, and these can be subdivided according to their cytokine secretion profile. One of these T cells, Th1 cells, secrete interferon gamma (IFN-γ) and exhibit protective effects against intracellular bacteria [21]. In contrast, Th17 cells function to protect against extracellular bacteria and fungi by secreting interleukin-17 (IL-17) A/F and IL-22. In addition, Th2 cells not only produce IL-4, IL-5, and IL-13, but also make it possible to protect against parasitic infection, while follicular helper T cells (Tfh cells) produce IL-21, which promotes B cells to produce antibodies [22,23]. Regulatory T cells (Tregs cells) produce IL-10 and transforming growth factor beta (TGF-β), immunosuppressive cytokines that control the inflammatory process [24]. During the development of naive T cells, the programming to a subset of T cells is regulated by unique transcription factors such as T-box transcription factor (T-bet), GATA binding protein 3 (Gata3), B-cell lymphoma 6 protein (Bcl-6), forkhead box P3 (FoxP3), and retinoic acid-related orphan receptor gamma t (RORγt) [21,25]. Increased levels of cytokines and an increase in the number of specific T cell subsets in the skin are associated with inflammatory reaction patterns and skin diseases [26]. For each example, an increase in Th17-derived cytokine is associated with the psoriasis response pattern of epidermal tissue, and Th1-derived cytokine IFN-γ is associated with the cytotoxicity and vacuole degeneration of basal keratinocytes (i.e., lichenoid response pattern). Meanwhile, Th2-derived cytokines are associated with spongiform formation (i.e., eczematous response pattern) in the epidermis, and Tfh cells activation is associated with antibody-mediated bullous skin disease.

So far, we have reviewed the cellular responses and the products that occur in inflammatory skin diseases. Based on the immunological principles discussed above, the application of metformin to several well-known representative inflammatory skin diseases and its molecular mechanisms of action will be reviewed in detail.

## 4. Psoriasis

Psoriasis is a common chronic inflammatory skin disorders characterized by hyperproliferation of keratinocytes and inflammation. Psoriasis is generally considered as a lifelong disease with notable morbidity [27]. The prevalence of psoriasis reported worldwide ranges from 0.09% to 11.43%, thus it is a serious health problem that affects at least 100 million people. Psoriasis is associated with the skin and nails and it is linked to several comorbidities. Skin lesions are localized or generalized with distinct red papules and plaques, usually covered with white or silver scales. Most of all, the lesion causes itching, tingling and pain. Several new biological and non-biological drugs with optimal effectiveness and sufficient safety are now available for psoriasis. These new treatments have immune modulating rather than immune suppressing actions and have overcome the efficacy limitations and safety issues of previous treatments [28,29,30]. Thus, research to develop effective, safe, and low-cost treatments is being conducted in various fields [31,32].

Psoriasis patients tend to have an increased risk of developing new diabetes, and on the contrary, patients with diabetes have an increased risk of developing psoriasis [33]. These results not only suggest a strong link between psoriasis and diabetes, but also suggest that the two diseases may share an inflammatory pathophysiology [34]. In terms of its association with diabetes, there is increasing evidence of the positive effects of anti-diabetic drugs on psoriasis including glucagon-like peptide-1 (GLP-1) receptor agonists, dipeptidyl peptidase-4 (DPP-4) inhibitors, thiazolidinedione and biguanide [35,36,37,38,39,40]. Therefore, it is important to summarize the clinical data that shows the anti-psoriatic effect of anti-hyperglycemic drugs and discuss their potential mechanisms of action. Through this, it is believed that a treatment can be identified that effectively treats psoriasis and diabetes without showing immune suppression-related side effects. A retrospective case-controlled study of the incidence of psoriasis in diabetic patients found that metformin treatment resulted in a statistically significant reduction in the incidence of psoriasis compared to those who did not receive metformin treatment [36]. These findings suggest that metformin therapy may lower the risk of developing psoriasis, at least in patients with diabetes. Another population-based study showed that frequent use of metformin reduced the incidence of new onset psoriasis in people with diabetes [41]. In addition, as a result of a single open-label randomized controlled trial, the incidence of psoriasis in patients treated with metformin was statistically significantly reduced compared to placebo in patients with concomitant metabolic disorders [42].

There are reports that suggest a potential molecular mechanism of action for metformin that has beneficial effects in the treatment of psoriasis. First, AMP-activated protein kinase has been shown to exert anti-inflammatory effects in macrophages [43]. It has also been reported that metformin treatment results in decreased inflammatory markers and cytokines such as tumor necrosis factor alpha (TNF-α) and IL-1β [44]. In the keratinocytes corresponding to the epidermal tissue, it has been found that metformin reduces cell proliferation through the mitogen-activated protein kinase (MAPK) signaling pathway [45]. More interestingly, it has been reported that metformin inhibits proliferation and pro-inflammatory cytokines (IL-6, TNF-α, and VEGF) through mammalian target of the rapamycin (mTOR) signaling pathway in cultured human keratinocytes [46]. Considering that the activation of Th17 and Tregs plays an important role in psoriasis, it is also worth noting that metformin reduced the percentage of Th17 cells and increased the percentage of Treg cells [47].

As discussed above, it was confirmed that the action of metformin in the treatment of psoriasis can occur through the alleviation of hyperglycemic conditions, and also through the reduction of various inflammatory cytokines. However, additional clinical studies are essential to confirm the feasibility and safety of metformin therapy in psoriasis patients with or without metabolic disorders.

## 5. Acanthosis Nigricans

Acanthosis nigricans is a skin condition that is characterized by dark, velvety discoloration of folds and wrinkles of the body. In most cases, acanthosis nigricans affects the armpits, neck and groin, and the affected skin may thicken [48]. Skin changes due to acanthosis nigricans usually occur in people with obesity or diabetes. Especially, children who develop acanthosis nigricans are at a higher risk of having type 2 diabetes. In rare cases, acanthosis nigricans can be a warning sign of cancers in certain organs including the stomach and liver [49].

Even though there is no specific treatment for acanthosis nigrican, treating the underlying condition can restore the normal color and texture of the affected skin areas, at least in part. Recently, there has been an increase in interest in the benign symptoms of acanthosis nigrican, which appear to be associated with hyperinsulinemia and insulin resistance [50,51,52]. Insulin binds to specific cell surface receptors and functions as a growth factor for keratinocytes and fibroblasts. The beneficial effects of metformin in alleviating disease states such as hyperinsulinemia or acanthosis nigricans are probably mediated, at least in part by its effect on glucose transporter type 4 (GLUT4) [53]. Interestingly, a TBC1D4 mutation (heterozygous premature stop mutation) was found in patients with both hyperinsulinemia and acanthosis nigrican, and it has been reported that this causes problems with GLUT4 translocation in response to insulin [54]. Under normal conditions, insulin enhances glucose uptake into adipose cells and muscle cells by inducing the movement of vesicles that contain GLUT4 from intracellular space to the plasma membrane. Consistent with these results, in chronic insulin resistance or hyperinsulinemia conditions such as obesity and polycystic ovary syndrome (PCOS), endocytosis of GLUT4 is induced, GLUT4 translocation to the plasma membrane is inhibited, and GLUT4 expression is reduced in target tissues [55]. According to a previous study, AMPK is involved in GLUT4 translocation to membranes by metformin and Cbl/CAP plays a role in AMPK-mediated GLUT4 translocation [56]. When patients with juvenile acanthosis nigricans associated with obesity and insulin resistance received metformin treatment, it was reported that acanthosis nigricans was completely cured, and this clinical result seems to be due to the normalization of hyperinsulinemia [57]. In other words, it is reasonable to assume that the main mechanism of action of metformin in acanthosis nigricans is due to the anti-hyperinsulinemia effect rather than the antihyperglycemic effect.

The most impressive aspect of these findings is that long-term treatment with metformin led to a complete improvement of acanthosis nigricans in adolescents within the normal weight range.

Based on these results, it was concluded that obesity-related and insulin-dependent juvenile acanthosis nigricans is a reversible disease state that can be controlled to some extent through a combination of appropriate diet and metformin use.

## 6. Acne

Acne is one of the most common chronic inflammatory disease of the pilosebaceous follicles [58,59,60]. Various factors contribute to the pathogenesis of acne including increased sebum production, alteration in the quality of sebum lipids, follicular hyper-keratinization, local inflammatory response, proliferation of Cutibacterium acnes, and dysregulation of the hormone function [59,61,62]. It is well known that hyperandrogenism plays a pivotal role in acne by inducing acne or increasing its severity [59]. Androgen is a key factor in increasing the size of the sebaceous gland, promoting sebum secretion, and stimulating abnormal desquamation of the keratinocytes in the pilosebaceous unit [63,64]. Ovarian overproduction of androgens is believed to be due to two factors. The first is the increased insulin stimulation from ovarian steroid-secreting cells due to insulin itself as well as insulin-stimulated growth factors, such as insulin-like growth factor-1 (IGF-1) [59,65]. The other factor is decreased IGF binding protein activity [59]. In a previous study, serum IGF-1 levels showed a positive correlation with mean facial sebum excretion in post-adolescent patients with acne [61]. Cappel et al. suggested that serum levels of dihydrotestosterone and dehydroepiandrosterone sulfate also correlate with serum IGF-1 levels in adult women with acne [66]. Furthermore, serum IGF-1 deficiency has been shown to prevent the occurrence of acne [67]. These findings strongly support that elevated level of IGF-1 is closely related with the development of acne.

There has been growing concerns about the risks and side effects (such as irritation, bacterial resistance, and systemic toxicity) of standard therapies for acne [68] that use antibiotics and isotretinoin [9]. To overcome this challenge, various studies have been undertaken and the results have suggested novel therapeutic options for acne [69,70]. Among these, metformin has proved to be a potential therapeutic agent for acne, especially for women with PCOS. PCOS is the most well-known cause of hyperandrogenism in women, which usually involves acne, hirsutism, and menstrual disorder [60,71]. Metformin activates AMPK, increases insulin sensitivity, decreases IGF-1 levels, lowers androgenic hormone levels from the adrenal glands and ovaries, improves ovarian and functional adrenal hyperandrogenism in PCOS, and prevents the development of acne [58,60].

Kolodziejczyk et al. [72] prospectively studied the effects of 12 weeks of metformin therapy with 500 mg three times per day on 39 women with PCOS. Decreased levels of insulin, total and bioavailable T led to significant improvement of hyperandrogenism. Metformin treatment decreased the acne score by 14% (*p* < 0.0005) and improved hirsutism and menstrual regularity. Sharma et al. [60] evaluated the efficacy and safety of metformin in reducing acne severity in female patients with PCOS. Forty women with PCOS and comorbid acne were treated with metformin 500 mg three times a day for eight weeks. At the end of the treatment, acne load was significantly reduced (*p* < 0.001) and acne severity also showed a significant decrease at week 3 that was sustained through week 6 and 8 (*p* < 0.001). When compared with isotretinoin which is a systemic standard therapy for acne, metformin showed superior efficacy on intractable and late onset acne [73]. Seventy women with late-onset acne or acne resistant to common therapies were randomized and treated with metformin 500 mg twice daily or isotretinoin 20 mg every other day for 6 months. Metformin was more effective in decreasing the acne grade in patients with PCOS (*p* < 0.05). Lipid profile and fasting blood sugar were also improved in the metformin group, while the levels of liver enzymes and bilirubin were increased in the isotretinoin group. Robinson et al. proved the efficacy of metformin as an adjunct therapy for the treatment of acne [9]. Eighty-four patients were randomized to receive 250 mg of oral tetracycline twice daily and 2.5% topical benzoyl peroxide once daily with or without 850 mg of metformin daily for 12 weeks. The metformin group showed an increase in treatment success rates (*p* = 0.04) and reduced total lesions from baseline (*p* = 0.278), which suggests that metformin is an effective adjunct therapy for acne. Additionally, the efficacy of metformin has also been reported in the treatment of males with acne [74]. Twenty male subjects with an altered metabolic profile were enrolled and randomized as two groups: 10 patients were treated with 500 mg of metformin twice daily, in association with a hypocaloric diet (1500–2000 kcal) for 6 months (group A), while 10 patients did not receive any treatment with metformin or changed diet (group B). After 6 months of metformin treatment, group A had a statistically significant decrease in acne severity (*p* < 0.03), whereas group B showed no significant decrease. Insulin sensitivity was also improved in group A suggesting the possible use of metformin and diet as a potential adjuvant therapy for male patients with acne.

## 7. Hidradenitis Suppurativa

Hidradenitis suppurativa (HS) is an intractable chronic inflammatory disorder that affects around 2% of the population, typically young adult women [75,76,77]. It is often characterized by painful, recurrent, deep-seated, inflamed lesions in the flexural apocrine gland-bearing areas, such as the axillaries, perineum, inguinal, anogenital, sub-mammary and infra-mammary regions [75,76]. The etiology of HS remains enigmatic [78], however, a genetic factor seems to be the initial causative factor leading to dilatation and distortion of the upper infundibular tract, which is followed by occlusion and subsequent rupture, bacterial infection, re-epithelialization, formation of sinus tracts, fistulas, and scarring [58,79].

Topical or systemic antibiotics are often chosen as the first treatment option of HS. For more extensive or therapy-resistant cases, biologicals, such as infliximab or adalimumab, may be the next therapeutic option [80]. Although antibiotics decrease the inflamed lesions, the outcome is still unsatisfactory and the recurrence rate is high after drug discontinuation [80,81].

Recent studies have proven that there is a significant association between HS and PCOS [82,83,84,85]. Phan et al. analyzed five case-control studies comparing patients with HS versus non-HS by performing a systematic review and meta-analysis. From the pooled data of five case-control studies, the authors established that there was a significantly higher proportion of PCOS in HS cases compared with non-HS cases (*p* < 0.00001), therefore suggesting that HS patients with signs of hyperandrogenism may potentially benefit from anti-androgen treatment [86].

Metformin was suggested as a novel potential treatment option since it may act as a mild anti-androgen. In addition, metformin improves glucose utilization by increasing receptor sensitivity, which leads to reduced insulin resistance and hyperinsulinemia [87]. Since a number of HS patients suffer from low glucose tolerance [75], metformin provides another advantage in the treatment of HS.

Arun et al. first reported a case of HS not associated with PCOS that was effectively treated with metformin [88]. The patient had long-standing HS with type 2 diabetes mellitus and had been taking 500 mg of metformin three times daily over 5 years. During medication, HS maintained stable with improved glycemic control, however, after discontinuation of metformin, the patient experienced an HS flare. As a study, the patient was restarted on metformin 500 mg once daily for 3 months, which was increased to 1 g per day. Four months after metformin initiation, the patients’ HS was improved markedly without any side effects and their glycemic control was excellent.

Jennings et al. performed a retrospective chart review of 53 HS patients who were treated with metformin over 12 months [89]. Metformin was started at 500 mg once daily as a monotherapy and increased to twice daily after 2 weeks, if patients were able to tolerate this. Further dose increases were made depending upon the response and tolerability of the patient. The mean treatment duration time and mean dose were 11.3 months and 1.5 g per day, respectively. Metformin was well tolerated and subjective clinical response was seen in 68% with 19% of these having quiescent disease.

Metformin was also proved to be effective for HS when treated with liraglutide, levonorgestrel-ethinyl estradiol, dapsone, and finasteride [90]. A 19-year-old female patient had HS with PCOS, obesity, and fatty liver. Flares effectively decreased and no new lesion was found for 6 months. This patient utilized this regimen for 3 years.

In addition, metformin turned out to be safe in pregnant women with PCOS when treated to minimize intrapartum complications [91,92,93]. Metformin showed minimal excretion into the breast milk and no adverse effects on breastfed infants have been reported [94,95].

## 8. Allergic Contact Dermatitis

Allergic contact dermatitis is a common skin disease caused by type IV hypersensitivity to chemicals and biotics inducing eczematous lesions. It is well-known that when 2,4-dinitrofluorobenzene (DNFB), a skin sensitizer, is repeatedly applied topically, it causes ACD which is mediated by CD4+ T cells, CD8+ T cells, dendritic cells, and mast cells, and it also changes the skin cytokine production environment [96]. In addition to these cells, macrophages have been reported to play an important role in the development of ACD [97]. It was found that macrophages were recruited to the lesion site of ACD [98]. Other reports have shown that the production of inflammatory cytokines and chemokines from macrophages is involved in the process of ACD [97]. In view of the role of immune cells in inflammatory skin diseases discussed above, it is concluded that the main mechanism of action of metformin in ACD is to suppress the production of various inflammatory cytokines due to AMPK activation.

From a slightly different perspective, a paper that studied the positive effects of metformin in the treatment of ACD has suggested an interesting molecular mechanism of action. In addition to the anti-inflammatory mechanisms that have been investigated in several other diseases, the mechanism in which autophagy is partially involved was suggested [99]. In this paper, the authors indicated that metformin inhibited macrophage activation and consequently improved ACD through improved autophagy flux. The activity of the NLRP3 inflammasome is negatively regulated by autophagy. Autophagy proteins have been reported to regulate NLRP3-dependent inflammation by preserving mitochondrial integrity [100]. As a result, by confirming the inhibition of NOD-like receptor pyrin domain-containing protein 3 (NLRP3) inflammasome by metformin, a new mechanism of action of metformin that can connect ACD-inflammation-autophagy to one axis was discovered.

## 9. Conclusion and Future Perspectives

Metformin is an antidiabetic drug with a very wide range of pharmacological properties. In this review, we have summarized information on the beneficial effects of metformin on inflammatory skin diseases including psoriasis, acanthosis nigricans, acne, hidradenitis suppurativa, and allergic contact dermatitis. Based on this, the potential mechanisms of action for each effect have been estimated. In fact, it has been reported that metformin has a beneficial effect on other skin diseases not mentioned in this review, namely, non-inflammatory skin diseases (i.e., eruptive xanthomas, squamous cell carcinoma (SCC), melanoma), and the mechanism for this has not been fully elucidated yet. Therefore, in addition to the well-known classical mechanism of action of metformin, that is, its anti-hyperglycemic action, the AMPK activation/NF-kB inhibition process and the resulting altered interaction of the complex cytokine network make it a very promising option for the treatment of skin diseases in the future. Additionally, further studies on new regulatory mechanisms, including autophagy and antioxidant processes, will broaden the scope of skin diseases that can be effectively treated with metformin and ensure it is used accurately and safely. In order to determine the specific mechanism of metformin, it would be a helpful to compare the results of other type 2 diabetes treatments such as GLP-1 receptor agonists or DPP-4 inhibitors.

## Figures and Tables

**Table 1:** The potential mechanisms of action of metformin in inflammatory skin disorders.

Inflammatory Skin Disorders	Major Action	Potential Mechanism of Action
Psoriasis	Reduction of hyperglycemia	Improving glucose utilization
	Decrease of inflammatory cytokines	Activating AMPK
Acanthosis nigricans	Reduction of hyperglycemia	Stimulating GLUT4 translocation
Acne	Reduction of hyperandrogenism	Lowering IGF-1 and androgens
Hidradenitis suppurativa	Reduction of hyperandrogenism	Reducing androgen overproduction
	Reduction of hyperglycemia	Improving glucose utilization
Allergic contact dermatitis	Decrease of inflammatory cytokines	Activating AMPK
	Reduction of NLRP3 inflammasome	Improving autophagy flux

## Abbreviations

AMPK	5′-adenosine monophosphate-activated protein kinase
GLP-1	glucagon-like peptide-1
DPP-4	dipeptidyl peptidase-4
mTOR	mammalian target of the rapamycin
GLUT4	glucose transporter type 4
PCOS	polycystic ovary syndrome
IGF-1	insulin-like growth factor-1
HS	hidradenitis suppurativa
ACD	allergic contact dermatitis
NLRP3	NOD-like receptor pyrin domain-containing protein 3
NF-κB	nuclear factor kappa-light-chain-enhancer of activated B cells
11β-HSD1	11β-hydroxysteroid dehydrogenase type 1
PARP-1	poly [ADP-ribose] polymerase 1
SIRT1	NAD-dependent deacetylase sirtuin-1
